# NIR‐Responsive ZIF‐8 Metal‐Organic Framework Nanohybrids with Photothermal, Antimicrobial, and Osteoinductive Properties to Prevent Implant Infection

**DOI:** 10.1002/mabi.202400594

**Published:** 2025-02-27

**Authors:** Cho‐E Choi, Yasmeen Shamiya, Wei Luo, Arghya Paul

**Affiliations:** ^1^ Department of Chemical and Biochemical Engineering The University of Western Ontario London ON N6A 5B9 Canada; ^2^ Department of Chemistry The University of Western Ontario London ON N6A 5B9 Canada; ^3^ School of Biomedical Engineering The University of Western Ontario London ON N6A 5B9 Canada; ^4^ Department of Chemical and Biochemical Engineering School of Biomedical Engineering Department of Chemistry The Centre for Advanced Materials and Biomaterials Research The University of Western Ontario London ON N6A 5B9 Canada

**Keywords:** antibacterial activity, bone repair, hydrogels, nanomedicine, stimuli‐responsive

## Abstract

Current treatments for bone injuries face notable limitations such as adverse reactions to implant materials and increased risks of infection. There is an essential need for a therapeutic that will address these issues and decrease recovery times. Herein, a multifunctional nanohybrid zinc‐based metal‐organic framework integrated with gold nanoparticles (Au@ZIF‐8) is synthesized to promote antibacterial and osteogenic benefits. Au@ZIF‐8 is capable of converting light energy into heat and has demonstrated its ability to increase the surrounding temperature by ≈30 °C. As a result, Au@ZIF‐8 has exhibited bactericidal activity against methicillin‐resistant Staphylococcus aureus (MRSA) upon exposure to near‐infrared (NIR) irradiation. Concurrently, Au@ZIF‐8 sustains the release of zinc ions from the nanohybrid for the potential of bone repair. When combined with a gelatin‐based hydrogel, Au@ZIF‐8 significantly elevated osteogenic gene expression and promoted preosteoclast differentiation through the sustained zinc ion release, as opposed to a gel‐only control. The potential of the multifunctional nanohybrid is further demonstrated as a coating material for titanium orthopedic implants to introduce antibacterial properties and promote osteogenic differentiation of preosteoblasts for bone healing. Given its excellent antibacterial in response to NIR irradiation and osteogenic abilities, Au@ZIF‐8 is a promising photothermal therapy for bone injuries.

## Graphical abstract

This paper presents the development of multifunctional nanohybrid structures consisting of zinc‐based metal‐organic frameworks and gold nanoparticles. These nanohybrid structures are photothermal in nature. They exhibit antimicrobial properties when exposed to NIR light. Additionally, they demonstrate inherent osteogenic properties by slowly releasing zinc mineral ions into the surrounding environment. Because of their multifunctional properties, these nanohybrids can be coated, alone or in combination with polymeric hydrogels, on bone implants to induce localized therapeutic effects.

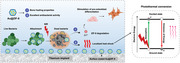

## 1 Introduction

Bone injuries, including fractures and breaks, are critical medical concerns due to their significant impact on mobility and overall quality of life. In 2019, there were 178 million new fractures reported worldwide, representing a 33.4% increase since 1990, according to a comprehensive study conducted by the WHO and coordinated by the Institute of Health Metrics and Evaluation (IHME).^[^ ^1^ ^]^ Such injuries can profoundly affect an individual's daily activities and well‐being, underscoring the need for prompt and effective management. Ensuring timely and effective treatment is crucial for restoring function and preventing complications that can arise from delayed or incomplete healing. Despite advancements in medical technology, the treatment of bone injuries remains a challenge, particularly in ensuring optimal healing and minimizing risks associated with surgical interventions and orthopedic implants.^[^ ^2^ ^]^ One major concern with orthopedic implants is the potential for adverse effects, especially those related to bacterial infections and lack of bioactive properties.^[^ ^2^, ^3^ ^]^ Post‐surgical infections pose a significant risk, often resulting in extended hospital stays, additional surgeries, or even treatment failure.^[^ ^3^, ^4^ ^]^ Among implant‐related infections, Staphylococcus aureus (S. aureus) is a major cause of bacterial infections and treatment failure, with methicillin‐resistant Staphylococcus aureus (MRSA) presenting an even greater threat due to its resistance to antibiotics.^[^ ^5^ ^]^ MRSA infections can lead to severe complications, including sepsis or death, making effective antimicrobial strategies crucial for implant success.^[^ ^6^, ^7^ ^]^ To manage these infections, high doses of antibiotics are often required, leading to systemic side effects and exacerbating the issue of antibiotic resistance. Additionally, conventional orthopedic implants, such as those made from titanium, lack the ability to deliver therapeutic agents and are typically devoid of the biofunctional properties necessary for effective biological interaction.^[^ ^8^, ^9^ ^]^ Their bioinert nature and lack of biological functionalization limit their capacity to engage with surrounding tissues, reducing their potential to promote bone regeneration and healing.^[^ ^9^, ^10^ ^]^ Bioactive agents are critical for stimulating cellular activity and tissue formation, yet traditional implants do not facilitate this biological interaction.^[^ ^8^, ^11^ ^]^ Recent studies have explored nanoparticles designed for either antibacterial activity or bone tissue regeneration, addressing the distinct challenges of preventing implant‐related infections and promoting osteogenesis.^[^ ^5^, ^12^, ^13^, ^14^ ^]^ However, significant challenges remain, including the precise control of bioactive release kinetics, potential cytotoxicity at higher concentrations, and maintaining a stable balance between antimicrobial efficacy and osteogenic stimulation, and the growing concern of antibacterial drug resistance. These limitations highlight the need for innovative and safe strategies to simultaneously tackle these complex issues effectively.^[^ ^15^ ^]^ Given these challenges, there is an urgent demand for innovative, drug‐free solutions that can prevent implant‐related infections, improve implant durability, and extend implant lifespan, ultimately benefiting patient outcomes.

One particularly promising nanoparticle is Zeolitic Imidazolate Framework‐8 (ZIF‐8), a type of metal‐organic framework (MOF) known for its excellent biocompatibility and ability to carry bioactive agents.^[^ ^10^ ^]^ ZIF‐8 not only allows for the sustained release of therapeutic agents, such as essential zinc ions and minerals, directly at the injury site but also significantly enhances bone repair processes.^[^ ^10^ ^]^ This integration is crucial, as it imparts vital biofunctionalities to the implant, enabling active interaction with the biological environment, and thereby promoting more effective bone healing and regeneration.^[^ ^10^ ^]^ Moreover, gold nanoparticles (AuNPs) are gaining significant attention for their potent antibacterial properties, particularly in photothermal therapy.^[^ ^15^ ^]^ The use of light, particularly near‐infrared (NIR) light, as an external stimulus has gained considerable attention in biomedical applications, including drug delivery, tumor therapy, and antibacterial treatments.^[^ ^16^, ^17^, ^18^, ^19^, ^20^, ^21^ ^]^ This non‐invasive approach leverages the strong optical properties of nanoparticles (e.g., Au, Pt, black phosphorus) to induce a photothermal effect through localized surface plasmon resonance (LSPR).^[^ ^15^, ^22^ ^]^ Upon exposure to NIR irradiation, AuNPs generate localized heat capable of effectively eradicating bacteria, reducing dependency on antibiotics, and minimizing infection risks.^[^ ^15^, ^23^ ^]^ These advanced materials not only address the shortcomings of traditional implants but also markedly improve therapeutic outcomes, reduce the risk of complications, and minimize the need for further surgical interventions. Therefore, the combination of AuNPs with ZIF‐8 to form Au@ZIF‐8 nanohybrid can represent a promising approach in the development of drug‐free medical implants.

In this study, we present a ZIF‐8‐based nanohybrid embedded with AuNPs, designed to enhance antibacterial activity and promote bone healing. The nanohybrid demonstrates potential as a coating for titanium implants, offering the combined benefits of antimicrobial protection, and bone regeneration support. By addressing persistent challenges of implant infections in orthopedic implants, this coating not only improves biological functionality but also extends effective therapeutic effects. Its optimized surface physicochemical properties further promote high bioactivity, making it a promising solution for improved implant performance and integration with surrounding bone tissue. By integrating AuNPs into a ZIF‐8 matrix, the nanohybrids not only ensure biofunctionality on titanium implants but also enable the sustained release of bioactive molecules that support cellular growth and differentiation, which are critical for effective bone repair. The nanohybrid's multifunctionality is demonstrated by its excellent osteogenic differentiation ability and antimicrobial efficacy without any commercial drug presence, driven by the release of zinc ions from the ZIF‐8 and the inherent antibacterial properties of the AuNPs. Additionally, the Au@ZIF‐8 composite can be stimulated by photothermal capabilities; when exposed to near‐infrared light, the AuNPs convert light into heat, resulting in a sustained, localized temperature increase that is lethal to bacteria while simultaneously promoting osteogenic activity. This synergistic effect not only improves the antimicrobial environment at the site of bone defects but also supports the osteoinductive processes that are vital for bone repair. This unique combination offers dual benefits: it ensures the sustained release of bioactive molecules to promote osteogenesis while improving the durability of the coated implant by minimizing degradation. Unlike previous studies that separately highlight the antibacterial activity of gold under photothermal therapy or the bone repair potential of ZIF‐8, this research is the first to integrate Au@ZIF‐8 for bone healing, leveraging photothermal therapy to enhance osteogenesis and overcome the limitations of current orthopedic implants. As a result, the Au@ZIF‐8 nanohybrid structure emerges as a highly promising solution for overcoming the current limitations of orthopedic implants including bacterial infection, overdose of antibiotics, and incomplete bone repair, with the potential to significantly enhance patient outcomes in bone injury treatment.

## 2 Material and Methods

Gold nanoparticles (5 nm diameter, Cat: 752 568), methanol, 2‐methylimidazolate (abbreviated to 2‐MeIm), and zinc nitrate hexahydrate (Zn(NO_3_)_2_ 6H_2_O), zincon spectrophotometric, chitosan (Cat: 448 869), dopamine hydrochloride (Cat: H8502), Minimum Essential Medium Eagle with alpha modifications (α‐MEM), fetal bovine serum (FBS), trypsin (0.25%), and ß‐glycerophosphate were purchased from Millipore Sigma, USA. The titanium alloy (Ti6Al4V) disks were 3D‐printed at the Additive Design in Surgical Solutions facility in London, ON, using a commercial AM400 3D metal printer from Renishaw plc, Wotton‐under‐Edge, Gloucestershire, UK. Power supply (model no. PSU‐H‐LED) with diode infrared laser module at 808 nm was obtained from Laserglow Technologies, USA.

### 2.1 Synthesis of Au@ZIF‐8 Nanohybrid

The Au@ZIF‐8 nanohybrid was synthesized using a modified version of the procedure described in the literature.^[^ ^24^ ^]^ The Au@ZIF‐8 nanohybrid was synthesized through the growth of the ZIF‐8 framework using zinc nitrate and 2‐methylimidazole (2‐Melm). During the formation process, gold nanoparticles were effectively entrapped within the ZIF‐8 structure. Briefly, the Zn^2^⁺:2‐Melm ratio was maintained at 1:2 to ensure optimal framework growth. To begin, 2 mL of AuNPs was added to an aqueous solution of zinc nitrate hexahydrate (Zn(NO₃)₂·6H₂O, 330 mg in 5 mL of water). This mixture was then slowly added dropwise to a solution of 2‐Melm (330 mg) dissolved in 10 mL of methanol. The reaction mixture was stirred continuously for 24 h at room temperature, resulting in the formation of a red precipitate. The precipitate was collected by centrifugation at 14000 rpm for 30 min. ZIF‐8 was synthesized without the addition of AuNPs.^[^ ^10^ ^]^ The resulting product was washed three times with methanol to remove any unreacted precursors and by‐products. Finally, the product was dried under ambient conditions and stored for further analysis.

### 2.2 Characterization of Au@ZIF‐8 Nanohybrid

Transmission electron microscopy (TEM) was employed to analyze the surface characteristics of synthesized Au@ZIF‐8 nanohybrid using a Philips 420 transmission microscope. The morphology of the Au@ZIF‐8 nanohybrid was observed via scanning electron microscopy (SEM) on a Hitachi SU8230 instrument at an acceleration voltage of 20 kV. The composition and elemental distribution within the samples were further analyzed through energy‐dispersive X‐ray spectroscopy using an X‐Flash 6160 EDX detector (Bruker, USA). Fourier Transformed Infrared (FTIR) spectra were obtained with a Nicolet Summit FTIR Spectrometer (Thermo Fisher, USA) to determine the chemical composition and functional structure of the samples at room temperature, ranging from 400 to 4000 cm^−1^. Optical properties were assessed using a SPARK multimode microplate reader UV–vis spectrophotometer (Tecan, USA), while the powder XRD patterns were analyzed using a Bruker Kappa Axis Apex2 (USA).

### 2.3 Photothermal Performance

A total of 1 mL each of AuNPs or Au@ZIF‐8 solution was transferred into a quartz cuvette and heated to 37 °C before being exposed to an 808 nm laser irradiation with 1 W cm^−2^. As a control, 1 mL of water was used. Each experiment was conducted in triplicate. The temperatures of the solutions were continually monitored using a FLIR One thermal imaging camera (Teledyne FLIR, USA). For the cycling measurements, the samples were irradiated for 5 min followed by natural cooling to room temperature.

### 2.4 Photothermal Conversion Efficiency

The photothermal properties of the Au@ZIF‐8 nanohybrid were assessed by irradiating a 2 mg mL^−1^ solution in water with NIR light at 808 nm and 1 W cm^−2^ for 5 min. Temperature measurements were recorded every 30 s during irradiation. For comparison, ZIF‐8 (2 mg mL^−1^) served as a control. Following the 5‐min irradiation period, the Au@ZIF‐8 solution was allowed to cool naturally, and its photothermal conversion efficiency (η) was subsequently calculated using the equation below (see details in the Supporting Information).^[^ ^25^, ^26^, ^27^ ^]^

(1)

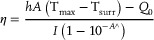




In this context, the variables are defined as follows: *h* represents the thermal conversion coefficient, while *A* denotes the surface area of the container. *T_max_* indicates the maximum temperature achieved, and *T_surr_* corresponds to the surrounding temperature of the container. *I* refer to the laser power, *A* represents the absorbance of the dispersion at 808 nm, and *Q_0_* accounts for the heat absorbed and dissipated by the solvent and container.

### 2.5 Zinc Release from Au@ZIF‐8 Nanohybrid Under NIR Irradiation

The release of zinc from the Au@ZIF‐8 nanohybrids (2 mg mL^−1^) were monitored using zincon spectrophotometric analysis (*n* = 5) following a previously established method.^[^ ^10^ ^]^ This technique involves reacting zinc ions with zincon dye to form a colored complex, which can be measured by absorbance at 620 nm. The intensity of the color, corresponding to the amount of zinc present, was used to quantify the zinc concentration in solution. The zinc release profile was tracked over time to assess the stability and controlled release properties of the nanoparticles in phosphate‐buffered saline (PBS, pH 7.4) at 37 °C. Additionally, the release was compared to a standard ZIF‐8 solution under both NIR irradiation OFF and ON conditions.

### 2.6 Bacterial Cell Culture

Methicillin‐resistant *Staphylococcus aureus* (MRSA) was chosen as the model bacterium for this study. The bacteria were cultured in tryptic soy broth at 37 °C with shaking at 250 rpm for 20 h. Afterward, the culture was centrifuged at 3000 rpm for 3 min. The resulting bacterial pellet was resuspended in PBS to a concentration of 1 × 10^8^ CFU mL^−1^ for subsequent experiments.

### 2.7 Assessment of Antibacterial Activity in Au@ZIF‐8 Nanohybrids

A 10 µL aliquot of the MRSA bacterial solution (1 × 10^6^ CFU mL^−1^) was mixed with 200 µL of Au@ZIF‐8 nanohybrids, AuNPs, and ZIF‐8 in a 96‐well plate and incubated at 37 °C for 30 min. The mixture was then exposed to NIR light for another 30 min at a distance of 3 cm After the reaction, the mixture was diluted tenfold with PBS, and a 100 µL sample was spread onto TSB‐agar plates. These samples were analyzed using the standard plate count method to assess bacterial cell viability. Bacterial viability was calculated by the following equation:

(2) $$\mathrm{Bacterial}\;\mathrm{viability}\left(\%\right)=\frac{\mathrm{CFU}\;\mathrm{of}\;\mathrm{sample}}{\mathrm{CFU}\;\mathrm{of}\;\mathrm{control}}\times 100\%$$

This equation is used to determine the percentage reduction of bacterial colony‐forming units (CFU) after treatment compared to an untreated control. The result gives the antibacterial efficacy of the treatment. Control groups were used for normalization.

### 2.8 Generating Au@ZIF‐8 Coated Titanium Implant

Titanium discs with a diameter of 24 mm and a thickness of 3 mm were cleaned in ethanol prior to use. To apply the Au@ZIF‐8 nanohybrid coating, a polydopamine (PDA) layer was deposited on the surface of the titanium discs under alkaline conditions. The discs were immersed in a dopamine solution (4 mg mL^−1^, 10 mm Tris‐HCl buffer, pH 8.5; Sigma–Aldrich, USA) and an Au@ZIF‐8 nanohybrid solution (2 mg mL^−1^) at 37 °C for 24 h. After coating, the Au@ZIF‐8‐coated discs were ultrasonically rinsed three times with deionized water to remove any unattached molecules, followed by drying with ambient air.

### 2.9 In Vitro Cytotoxicity of Au@ZIF‐8 Nanoparticles

The cytotoxic effects of Au@ZIF‐8 nanohybrids on MC3T3 pre‐osteoblast cells (Sigma–Aldrich, USA) were assessed. Cells were plated at a density of 5000 cells/well in a 96‐well plate and incubated under 5% CO_2_ at 37 °C. Various concentrations of Au@ZIF‐8 nanohybrids encapsulated in Gelatin Methacrylate (GelMA) hydrogels were introduced to the culture medium, followed by an additional 24‐h incubation period. Afterward, 10 µL of the MTS reagent (3‐(4,5‐dimethylthiazol‐2‐yl)‐5‐(3‐carboxymethoxyphenyl)‐2‐(4‐sulfophenyl)‐2H‐tetrazolium, Promega, USA) was added to each well and incubated for 2 h. The supernatant from each well was then collected and analyzed for absorbance at 490 nm using a microplate reader (The SPARK multimode microplate reader, Tecan, USA) to determine cell viability. Additionally, a cytotoxicity assay under NIR irradiation was performed using MC3T3 cells treated with ZIF‐8 and Au@ZIF‐8 nanoparticles (each at a concentration of 2 mg mL^−1^), followed by 5 min of irradiation and incubation under 5% CO₂ at 37 °C for 4 h.

### 2.10 Live/Dead Fluorescent Imaging

To evaluate the viability of cells exposed to Au@ZIF‐8 nanohybrids, a Live/Dead Cell imaging kit was employed (Thermo Fisher Scientific, USA, Cat: R37601) Briefly, 2 µL of a solution containing Calcein‐AM and Propidium Iodide (PI) was added to each well of a 24‐well plate. The cells were incubated for 20 min, after which the medium was replaced with fresh culture medium. Subsequently, the samples were analyzed using fluorescence microscopy (Nikon, Eclipse Ti2, USA). Calcein‐AM, a non‐fluorescent compound that permeates live cells, fluoresces upon metabolic activation. In contrast, PI, a fluorescent nucleic acid dye, selectively enters and stains cells with damaged membranes, indicating cell damage or death.

### 2.11 Osteogenic Differentiation Activity

#### 2.11.1 Biomineralization Through Alizarin Red S Staining

Alizarin Red S (ARS) staining was employed to evaluate the biomineralization of the extracellular matrix (ECM). MC3T3 cells were seeded onto ZIF‐8 and Au@ZIF‐8 nanohybrids encapsulated within a GelMA hydrogel and cultured in an osteogenic induction medium for 21 days. Following the culture period, the cells were fixed with 4% paraformaldehyde for 30 min. The hydrogels, with adherent cells, were then stained with Alizarin Red S solution (ARS, ScienCell Research Laboratories, ARS Staining Quantification Assay, USA) for 30 min at room temperature. After staining, the hydrogels were washed with deionized water until the rinse water was clear. Mineralized nodules were visualized using a Nikon Eclipse Microscope (Nikon, Canada) and processed using Nikon's NIS Elements software. For ARS quantification, 10% acetic acid was added to the stained cells, followed by incubation at 85 °C for 10 min. The mixture was then centrifuged at 20000 g for 15 min. The supernatant was transferred to new tubes and neutralized with 10% ammonium hydroxide. Subsequently, 150 µL of each sample was placed in a 96‐well plate, and the optical density at 405 nm (OD405) was measured using a Spark multimode microplate reader (Tecan, USA). The ECM mineralization assay was performed in quadruplicate to ensure reproducibility.

#### 2.11.2 Expression of Osteogenesis‐Related Genes

The expression levels of osteogenesis‐related genes were assessed using reverse transcription‐quantitative polymerase chain reaction (RT‐qPCR) after 21 days of cultivation. MC3T3 cells (1 × 10⁶) were seeded in a 24‐well plate and cultured in an osteogenic induction medium. Primer sequences used in the assay are listed in **Table** 1. The relative expressions of genes, including Runt‐related transcription factor 2 (Runx2), osteocalcin (OCN), osteopontin (OPN), and bone morphogenetic protein‐2 (BMP2), were quantified using the 2‐ΔΔCt method, with gene glyceraldehyde 3‐phosphate dehydrogenase (GAPDH) serving as the reference gene. The RT‐qPCR assays were performed in quadruplicate.

**Table 1:** RT‐qPCR primers used in this study.

Gene	Forward primer sequence (5′‐3′)	Reverse primer sequence (5′‐3′)	Ref
Murine OCN	AAGCAGGAGGGCAATAAGGT	TTTGTAGGCGGTCTTCAAGC	[28]
Murine OPN	AGCAAGAAACTCTTCCAAGCAA	GTGAGATTCGTCAGATTCATCCG	[29]
Murine Runx2	ACTCTTCTGGAGCCGTTTATG	GTGAATCTGGCCATGTTTGTG	[30]
Murine BMP2	ACACAGCTGGTCACAGATAAG	CTTCCGCTGTTTGTGTTTGG	[31]
Murine GAPDH	AGGTCGGTGTGAACGGATTTG	TGTAGACCATGTAGTTGAGGTCA	[32]

### 2.12 Statistical Analysis

Statistical data from quadruplicate tests were presented as mean ± standard deviation. The experimental results were analyzed using Prism 9 software (GraphPad, USA). One‐way ANOVA was employed to assess statistical significance for experiments involving more than two groups, such as cell proliferation studies using the MTS assay and RT‐qPCR analysis. Post hoc comparisons were conducted using the Tukey procedure, with a *p*‐value <0.05 considered statistically significant. A *p*‐value <0.05 was considered indicative of a significant difference and is denoted by ^*^.

## 3 Results and Discussion

### 3.1 Fabrication and Characterization of Au@ZIF‐8 Nanohybrid

**Figure 1:**
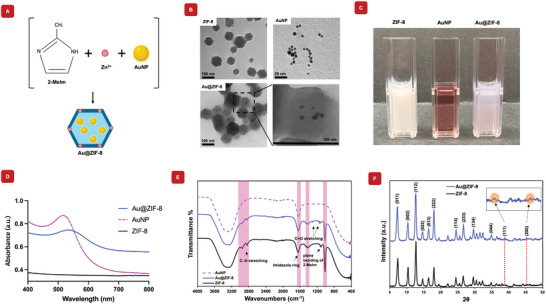
Characterization of Au@ZIF‐8 nanohybrids. A) Schematic illustration of the synthesis of Au@ZIF‐8 nanohybrid. B) TEM images of individual nanoparticles. C) Photograph of 1 mL samples in aqueous solution. D) UV–vis absorption spectra of ZIF‐8, AuNP, and Au@ZIF‐8. E) FTIR spectrum of ZIF‐8, AuNP, and Au@ZIF‐8. F) Powdered XRD pattern of ZIF‐8 and Au@ZIF‐8. This confirms the sodalite zeolite‐type crystal structure of the ZIF‐8 shell. The sharp, well‐defined peaks indicate a high degree of crystallinity. All prominent peaks, including 011, 002, 112, 022, 013, and 222, correspond precisely to those of pure ZIF‐8 crystals.

The synthesis of Au@ZIF‐8 nanohybrid involves encapsulating gold nanoparticles within ZIF‐8 to create a nanohybrid with unique properties. The synthesis process of the Au@ZIF‐8 nanohybrid, depicted in **Figure** **1**A, illustrates the step‐by‐step integration. The synthesis process of ZIF‐8 involves mixing zinc nitrate and 2‐methylimidazole in methanol, which allows the zinc ions to coordinate with the imidazole linkers to form the framework. During the formation of this framework, gold nanoparticles are introduced into the mixture, facilitating their encapsulation within the growing structure. The Au@ZIF‐8 nanohybrid was successfully synthesized and characterized using Transmission Electron Microscopy (TEM), as depicted in Figure 1B. The TEM images reveal that the nanohybrid possesses a well‐defined hexagonal structure, with particles consistently sized at ≈100 nm. Notably, when compared to ZIF‐8 alone, there is no significant difference in the shape or size of Au@ZIF‐8. This suggests that the AuNPs are effectively encapsulated within the ZIF‐8 framework, maintaining the original morphology of ZIF‐8. ZIF‐8 and AuNPs demonstrate excellent stability and dispersibility in aqueous solutions. As depicted in Figure 1C, the samples in aqueous solution exhibit distinct characteristics. Specifically, the Au@ZIF‐8 nanohybrid displays a light pink hue, confirming the successful formation of the composite. The UV–vis spectra of each sample, including the Au@ZIF‐8 nanohybrid, offer valuable insights into their optical properties, validating the successful synthesis of the material (Figure 1D). The photoabsorption properties of the aqueous dispersions were analyzed using UV–vis spectroscopy. At a concentration of 2 mg mL^−1^, ZIF‐8 showed no significant absorption within the 400–800 nm range, consistent with its characteristic lack of optical activity in the visible region. In contrast, the AuNPs exhibited a distinct peak at ≈540 nm, characteristic of their SPR. Entrapment of AuNPs within the ZIF‐8 framework resulted in an enhanced absorption profile for the Au@ZIF‐8 nanohybrid, particularly around the 540 nm region, attributed to the plasmonic behavior of the embedded AuNPs. Notably, the absence of significant spectral shifts or the emergence of additional absorption peaks compared to the individual spectra of AuNPs and ZIF‐8 indicates that the integration of AuNPs into the ZIF‐8 framework preserved their intrinsic optical properties. This observation confirms the structural integrity of both components and highlights the potential of Au@ZIF‐8 for applications reliant on stable optical properties. Fourier Transform Infrared (FTIR) spectroscopy was employed to characterize the Au@ZIF‐8 nanohybrid and to confirm the successful entrapment of AuNPs within the ZIF‐8 framework (Figure 1E). The FTIR spectrum of the nanohybrid shows characteristic absorption bands corresponding to the functional groups present in the ZIF‐8 structure, including the C═N stretching vibration ≈1580 cm⁻¹, C─H stretching ≈3130 cm⁻¹, and the imidazole ring vibrations ≈1350–1500 cm⁻¹. These bands are signatures of the imidazole linkers within the ZIF‐8 framework. Importantly, the FTIR spectra of the Au@ZIF‐8 nanohybrid closely resemble those of pure ZIF‐8, with no significant shifts or additional peaks. This indicates that the introduction of AuNPs does not alter the chemical structure of the ZIF‐8 framework. The absence of any new absorption bands suggests that there is no strong chemical interaction between the AuNPs and the ZIF‐8 framework, confirming that the AuNPs are physically encapsulated within the porous structure of ZIF‐8 rather than chemically bonded to it. These findings reveal that the ZIF‐8 framework remains intact after the incorporation of AuNPs, maintaining its structural integrity while successfully housing the nanoparticles. Powdered X‐ray diffraction (XRD) analysis was conducted to examine the crystalline structure of both ZIF‐8 and Au@ZIF‐8 nanohybrids. Figure 1F shows the powdered XRD analysis used to investigate the crystalline structure of both ZIF‐8 and Au@ZIF‐8 nanohybrids. The XRD spectrum of pure ZIF‐8 exhibits distinct diffraction peaks, characteristic of its highly crystalline and porous structure. The sharp, well‐defined peaks in the XRD spectrum correspond to specific planes within the ZIF‐8 crystal lattice, confirming the successful synthesis of ZIF‐8 with its characteristic hexagonal topology. In the case of the Au@ZIF‐8 nanohybrid, the XRD pattern retains the distinct peaks of the ZIF‐8 framework, indicating that the incorporation of Au nanoparticles does not disrupt the crystalline structure of ZIF‐8. Additionally, new diffraction peaks corresponding to the (111) and (200) planes of face‐centered cubic (fcc) gold (Au) appear in the Au@ZIF‐8 spectrum.^[^ ^33^, ^34^ ^]^ These peaks confirm the successful embedding of Au nanoparticles within the ZIF‐8 matrix while maintaining their crystalline nature. The comparison of the XRD spectra of ZIF‐8 and Au@ZIF‐8 demonstrates that the structural integrity of the ZIF‐8 framework is preserved after the incorporation of Au nanoparticles, with the additional peaks providing clear evidence of the presence of crystalline Au within the composite.

### 3.2 Photothermal Effects and Therapeutic Performance

**Figure 2:**
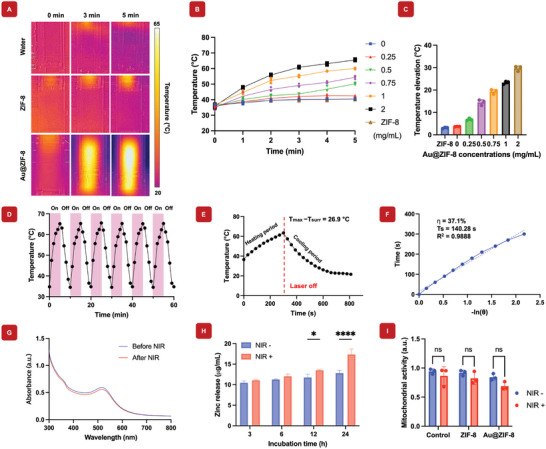
The evaluation of photothermal properties of Au@ZIF‐8 nanohybrid and cell cytotoxicity. A) Thermal images taken before and after 5 min of NIR irradiation show an increase in temperature when AuNPs are present. B) Photothermal effects start at 37 °C at different concentrations of Au@ZIF‐8 and are compared to 2 mg mL^−1^ of ZIF‐8 nanoparticles. (*n* = 3) C) The temperature elevation curve of Au@ZIF‐8 (2 mg mL^−1^) at different concentrations irradiated by 808 nm NIR laser for 5 min. (*n* = 3) D) Temperature changes of Au@ZIF‐8 under NIR irradiation over six ON/OFF cycles, demonstrating excellent photothermal stability throughout the cycles. E) Photothermal effect of the irradiation of the aqueous solution of the Au@ZIF‐8 (2 mg mL^−1^) with the NIR laser (808 nm, 1 W cm^−2^) started at 37 °C, in which the irradiation lasted for 800 s, and then the laser was shut off. F) Linear time data versus ‐ln(θ) obtained from the cooling period from E. G) UV–vis absorption of Au@ZIF‐8 before and after NIR irradiation for 5 min. H) The release amount Zn^2+^ from 2 mg mL^−1^ from Au@ZIF‐8 at different incubation times with or without NIR irradiation. I) *In vitro* MC3T3 cell cytotoxicity was evaluated with and without NIR exposure for 5 min. Although there were no significant differences within each group (NIR+ vs NIR‐), the data indicates that longer NIR exposure time may induce harmful effects to the cells and tissues. The dates are provided as the mean ± SD, *n* = 3 (^*^ *p* < 0.05, ^****^ *p* < 0.0001).

The photothermal behavior of Au@ZIF‐8 was investigated under 808 nm laser irradiation (1 W cm^−2^). As shown in **Figure** **2**A, the temperature profile of Au@ZIF‐8 nanohybrids in aqueous solution were measured using a disposable cuvette cell. In the presence of AuNPs, the temperature increased rapidly compared to the blank groups under NIR irradiation. Similarly, the dried samples exhibited a higher temperature increase in the AuNP‐containing groups (Figure S1, Supporting Information). The results demonstrated a positive correlation between the temperature increase of Au@ZIF‐8 and the duration of irradiation. To simulate the temperature response in a biological environment (≈37 °C), photothermal analysis was conducted at this temperature. Figure 2B shows the temperature response of various concentrations of Au@ZIF‐8 nanohybrids under NIR light irradiation. A clear trend was observed, higher concentrations of Au@ZIF‐8 nanohybrids resulted in greater temperature increases. Specifically, the temperature of the Au@ZIF‐8 nanohybrid at a high concentration of 2 mg mL^−1^ rose significantly, from 35.87 ± 1.15 to 65.53 ± 0.42 °C, within 5 min of NIR light irradiation at a distance of 3 cm. In contrast, the temperature of the blank group increased only slightly, from 35.0 ± 0.1 to 40.47 ± 0.15 °C. This indicates that the presence of AuNPs in ZIF‐8 greatly enhances the NIR‐induced temperature rise, highlighting the strong NIR response of AuNPs within the composite. The temperature elevation of Au@ZIF‐8 nanohybrids at varying concentrations under NIR light irradiation reveals a clear concentration‐dependent response, highlighting the nanohybrid's potential in photothermal therapy (Figure 2C). This indicates that temperature control can be precisely tuned by adjusting the concentration. Furthermore, as shown in Figure 2D, Au@ZIF‐8 demonstrates excellent photothermal stability, showing no temperature reduction over six cycles. Figure 2E showed the temperature increase after 300 s of irradiation, resulting in a temperature difference of 26.9 °C. Since photothermal conversion efficiency (η) is a key parameter for assessing the effectiveness of a photothermal therapy agent, the NIR‐photothermal conversion efficiency (η) of a 2 mg mL^−1^ Au@ZIF‐8 nanohybrid solution was measured under 808 nm laser irradiation at a power density of 1 W cm^−2^ using the Roper method.^[^ ^25^ ^]^ After irradiation, a natural cooling period was used to evaluate the photothermal conversion efficiency. The photothermal conversion efficiency of the Au@ZIF‐8 nanohybrid was determined to be 37.1% (Figure 2F), significantly higher than that of ZIF‐8 alone, which showed an efficiency of only 12.2% (Figure S2, Supporting Information). The unchanged absorption spectra after NIR irradiation further confirmed its robust photo‐ and chemical stability (Figure 2G). To assess the phototherapeutic potential of Au@ZIF‐8 nanohybrids, the release of zinc ions over 24 h, with and without NIR irradiation, was measured using the zincon spectrophotometric method (Figure 2H). In the absence of NIR irradiation, Zn ion release from ZIF‐8 was measured at 10.48 ± 0.43 µg mL^−1^ within the first 3 h, reaching 12.84 ± 0.67 µg mL^−1^ by 24 h. In contrast, under NIR irradiation, the release was significantly higher: 11.1 ± 0.2 µg mL^−1^ at 3 h, increasing substantially to 17.37 ± 1.28 µg mL^−1^ by 24 h. This indicates that NIR irradiation accelerates Zn ion release compared to non‐irradiated conditions. The accelerated release is attributed to the photothermal effect of NIR irradiation, which raises the local temperature and weakens the coordination bonds between Zn ions and the 2‐Melm ligands within the ZIF‐8 framework.^[^ ^35^, ^36^ ^]^ This elevated temperature facilitates faster degradation of ZIF‐8, resulting in a more rapid and pronounced release of Zn ions into the surrounding medium compared to the slow, gradual release observed under normal physiological conditions. These results indicate that NIR irradiation substantially enhances Zn ion release, with longer incubation times further promoting this effect. This suggests that NIR light and extended incubation induce the structural collapse of the nanohybrids, leading to increased Zn ions. These findings suggest that NIR light and prolonged incubation trigger the structural collapse of the nanohybrids, resulting in increased Zn ion release. The sustained degradation of ZIF‐8 further contributed to the release of gold nanoparticles, with longer incubation times leading to even greater Zn ion release. Thus, NIR light irradiation and extended incubation synergistically promote composite breakdown, thereby enhancing the overall release of therapeutic agents. Additionally, in vitro cell cytotoxicity assays confirmed comparable cell cytocompatibility following 5 min of NIR irradiation (Figure 2I and Figure S3, Supporting Information), although we observed a decreasing trend in mitochondrial activity when exposed to NIR. We acknowledge that the cytocompatibility of the nanohybrids under NIR irradiation can be further improved. Optimization of the exposure time, light intensity, and distance between the light and the cell culture will be done in future studies to ensure the highest biosafety of the material under NIR conditions.

### 3.3 Photothermal Properties of Au@ZIF‐8‐Coated Titanium Implants

**Figure 3:**
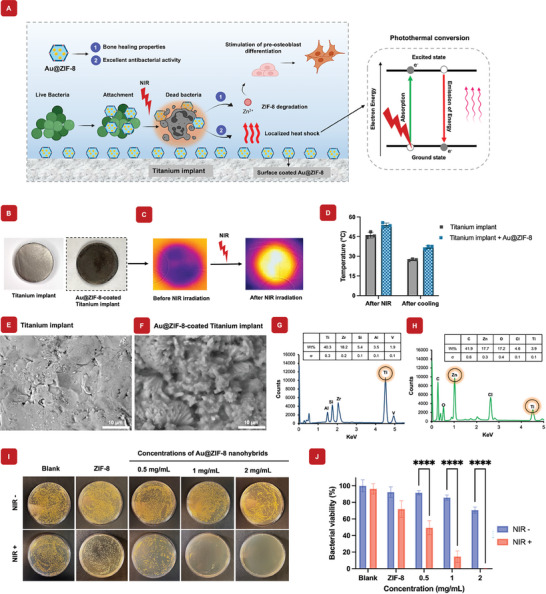
Photothermal antibacterial activity of Au@ZIF‐8 nanohybrid. A) Illustration depicting the antibacterial activation mechanism and osteogenesis stimulation process on Au@ZIF‐8‐coated titanium substrate under NIR irradiation. NIR irradiation triggers the photothermal conversion of the Au@ZIF‐8 nanohybrid, causing localized heating and ZIF‐8 degradation. This process enhances antibacterial effects by disrupting bacterial cell membranes. Simultaneously, the released zinc ions from the degraded ZIF‐8 promote osteogenic differentiation of surrounding cells, supporting bone tissue regeneration and mineralization on the titanium substrate. B) The optical images compare the Titanium implant before and after coating with Au@ZIF‐8, demonstrating a noticeable visual difference indicative of successful nanoparticle deposition. C) Thermal images of the Au@ZIF‐8‐coated Titanium implant under 5 min of NIR irradiation reveal a significant temperature increase, confirming the photothermal properties of the Au@ZIF‐8 coating. D) Temperature changes after 5 min of NIR irradiation and subsequent 5 min of cooling demonstrate that the Au@ZIF‐8 coated Titanium implant retained heat more effectively, suggesting their potential for enhanced therapeutic activity. E) and F) SEM images of Au@ZIF‐8‐coated titanium implants, without and with PDA coating respectively. G) and H) display the corresponding EDX analyses graph exhibiting distinctive Zn (coming from Au@ZIF‐8) and Ti peaks (coming from titanium implant). I) Photographs of MRSA bacterial colonies treated with a PBS blank, ZIF‐8, and varying concentrations of Au@ZIF‐8 nanohybrids were analyzed using the plate count method, both under NIR irradiation for 30 min and without light exposure in the dark. Au@ZIF‐8 nanohybrids demonstrated increased antibacterial effectiveness with higher concentrations. J) The relative bacterial viabilities were measured following treatment with a PBS blank, ZIF‐8, and Au@ZIF‐8, both under 30 min of NIR irradiation and in the dark. The PBS control in the dark was used as a reference for 100% cell viability. A value of ^****^ *p* < 0.0001 indicated a highly significant difference (*n* = 3).

Bone implant‐associated infections are a major concern in orthopedics and can lead to implant failure. For clinical aspect, coating surfaces with biocompatible nanoparticles can enhance bone formation and inhibit bacterial infections.^[^ ^37^ ^]^ The incorporation of AuNPs into ZIF‐8 (Au@ZIF‐8) has the potential to enhance the functionality beyond their individual roles. Under NIR light exposure, the photothermal properties of AuNPs promote antimicrobial activities, while concomitantly it promotes degradation of the ZIF‐8 component of the Au@ZIF‐8. This, in turn, enables controlled and localized release of zinc ions to promote osteogenesis. To assess the efficacy of synthesized Au@ZIF‐8 nanohybrids under NIR light irradiation, they were deposited onto titanium discs using PDA as an adhesion layer, ensuring better attachment of the nanohybrids to the titanium surface. **Figure** **3**A highlights the ability of Au@ZIF‐8 ‐coated nanohybrids to generate localized heat under NIR irradiation, demonstrating their photothermal performance and effectiveness in promoting antibacterial activation and osteogenesis stimulation. Figure 3B,C shows photographs of the titanium implant and both temperature readings before and after NIR light irradiation for the control and Au@ZIF‐8‐coated titanium implant. For further verification of the Au@ZIF‐8‐coated titanium implant, FTIR spectroscopy was performed, confirming the successful conjugation of PDA to Au@ZIF‐8 (Figure S4, Supporting Information). After 5 min of NIR light exposure, the temperature of the Au@ZIF‐8‐coated titanium implant significantly increased from 21.48 ± 0.9 to 53.76 ± 1.68 °C, compared to the control group, which increased from 20.53 ± 0.8 to 46.13 ± 2.21 °C. Notably, for 5 min of cooling, the Au@ZIF‐8‐coated titanium implant retained a temperature up to 36.9 ± 1.12 °C, while the control group's temperature dropped to 27.33 ± 0.6 °C, highlighting their potential for enhanced therapeutic activity (Figure 3D). Figure 3E,F presents SEM images of PDA‐uncoated and PDA‐coated titanium implants, with PDA‐coated titanium implants showing the distinct surface morphology changes when loaded with Au@ZIF‐8 nanoparticles. Figure 3G,H display the corresponding EDX spectra of Au@ZIF‐8 nanoparticles loaded PDA‐coated implants, confirming the elemental composition and verifying the successful deposition of the PDA coating on the titanium surface. Although this result demonstrates the beneficial effect of ZIF‐8 coating on implants, careful optimization of the nanoparticle loading concentration is essential. Higher concentrations of nanoparticles may lead to signific toxicity in the surrounding tissue environment, which could hinder the overall therapeutic outcomes. As an alternate, these Au@ZIF‐8 nanoparticles can be coated on the implants in combination with hydrogels. This may allow us to load higher concentrations of Au@ZIF‐8 to achieve superior therapeutic efficacy but with limited side effects. Overall, this prolonged heat retention and enhanced photothermal response of the Au@ZIF‐8‐coated titanium disc highlights its potential as a promising biomaterial for applications that require sustained antibacterial actions.

### 3.4 Photothermal Antibacterial Effects of Au@ZIF‐8 Nanohybrids

The photothermal‐induced antibacterial activities of Au@ZIF‐8 nanohybrids were investigated under NIR irradiation. Au@ZIF‐8 nanohybrids bind to bacterial cell walls through interactions with carboxyl groups present on the surface, leading to tight attachment and subsequent inactivation.^[^ ^38^, ^39^ ^]^ Moreover, the positively charged ZIF‐8 interacts electrostatically with the negatively charged carboxyl groups on the bacterial cell surface, facilitating strong adhesion. This disrupts the cell wall structure and triggers denaturation of membrane proteins.^[^ ^38^ ^]^ Upon irradiation with NIR light, the nanohybrids efficiently absorb the energy and convert it into localized heat, significantly raising the temperature in the vicinity of the bacterial cells. This thermal increase causes irreversible damage to the bacterial membranes and proteins, ultimately leading to their destruction.^[^ ^3^ ^]^ As demonstrated in Figure S5 (Supporting Information), the photothermal antibacterial activity was assessed by exposing an aqueous solution of Au@ZIF‐8 nanohybrids to NIR light. The bacterial cells were either treated with Au@ZIF‐8 nanohybrids under NIR irradiation or left untreated. The antibacterial activities of Au@ZIF‐8 nanohybrids were assessed by using a CFU counting assay as shown in the bacteria colony images in Figure 3I. Figure 3J showed the corresponding analysis of bacterial cell viability under these different conditions. Additionally, the partial cell death induced by ZIF‐8 under NIR‐off conditions may be attributed to the inherent antibacterial properties of ZIF‐8. The release of Zn^2^⁺ ions and rapid generation of reactive oxygen species (ROS) from ZIF‐8 are likely responsible for bacterial inhibition and cell death.^[^ ^40^, ^41^, ^42^ ^]^ As the concentration of Au@ZIF‐8 was increased to 0.5, 1, and 2 mg mL^−1^, the cell viability of MRSA under NIR irradiation decreased to 49.48 ± 8.3%, 14.6 ± 6.9%, and 0.25 ± 0.25%, respectively. This indicates that Au@ZIF‐8 significantly inhibited bacterial growth, achieving nearly complete eradication after 30 min of NIR light exposure. In contrast, Au@ZIF‐8 nanohybrids without NIR light irradiation resulted in partial cell death, with cell viabilities of 91.58 ± 2.58%, 85.73 ± 3.2%, and 70.7 ± 3.7%, when exposed to 0.5, 1, and 2 mg mL^−1^ Au@ZIF‐8, respectively. This suggests that the intrinsic antibacterial properties of AuNPs and ZIF‐8, including the bactericidal action of zinc ions from ZIF‐8, contribute to the observed effects. The partial cell death observed with Au@ZIF‐8 is likely due to the intrinsic antibacterial properties of zinc ions released during the degradation of ZIF‐8, which effectively inhibit and kill bacteria.^[^ ^19^ ^]^ Here we have used a range of 0 to 2 mg ml^−1^ of Au@ZIF‐8 nanoparticles to test their in vitro cytocompatibility and antibacterial properties. However, for clinical applications, further optimization of NIR exposure conditions will be required to determine safe thermal thresholds and prevent damage to surrounding tissues.^[^ ^43^ ^]^ Overall, these results highlight the strong photothermal antibacterial effects of synthesized Au@ZIF‐8 nanohybrids, emphasizing their potential to be used in clinical settings where antibiotic‐resistant bacterial infections is an ongoing concern.

### 3.5 In Vitro Cytotoxicity of Au@ZIF‐8 Nanoparticles within Hydrogels

**Figure 4:**
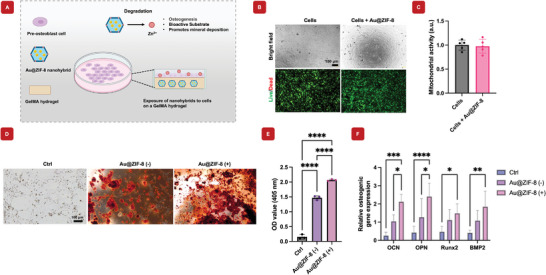
Osteogenic differentiation and *In vitro* cytocompatibility of Au@ZIF‐8 nanoparticles in hydrogels. A) Schematic visualization of the method by which the cells were exposed to the Au@ZIF‐8 hydrogels. B) Bright field and fluorescence live/dead cell images of pre‐osteoblast cells, showing excellent cell biocompatibility. C) Cell viability of pre‐osteoblast cells evaluated by MTS cell proliferation assay, showing no adverse effect on cells when Au@ZIF‐8 in the nanohybrid hydrogels (*n* = 5). D) Alizarin Red staining was used to compare the Ctrl, Au@ZIF‐8 (‐), and Au@ZIF‐8 (+), revealing increased mineral deposition in the Au@ZIF‐8‐treated groups. E) The corresponding ARS optical density value at 405 nm analysis revealed a significant increase in calcium deposition in the Au@ZIF‐8‐treated groups. F) RT‐qPCR analysis revealed that the presence of ZIF‐8 significantly upregulated osteogenic‐related genes, including OCN, OPN, Runx2, and BMP2, underscoring its effectiveness in promoting bone regeneration. A value of ^*^ *p* > 0.05 indicated no significant difference, a value of ^**^ *p* < 0.01 indicated a significant difference, ^***^ *p* < 0.001 ^****^ *p* < 0.0001 indicated a highly significant difference. (*n* = 3).

To determine the optimal biocompatible concentration of Au@ZIF‐8 nanohybrids, an in vitro cell viability analysis was performed. Previously, we optimized ZIF‐8 at a concentration of 2 mg mL^−1^ within the hydrogel to demonstrate its high cell cytocompatibility. Therefore, additional cytotoxicity experiments for ZIF‐8 were not conducted in this study.^[^ ^10^ ^]^ The Au@ZIF‐8 nanohybrids were encapsulated within polymeric GelMA hydrogel networks to improve both the biocompatible and structural supports of the nanohybrids. These nanohybrids were then cocultured with preosteoblast cells to assess their biocompatibility and potential for promoting cell growth (**Figure** **4**A). After 24 h of culture, cell viability was assessed using Live/Dead imaging. Both bright‐field and fluorescence images of live cells demonstrated excellent viability up to a concentration of 2 mg mL^−1^ of Au@ZIF‐8 nanohybrids (Figure 4B). Additionally, mitochondrial activity assays of the preosteoblast cells indicated no cytotoxic effects at concentrations up to 2 mg mL^−1^ of Au@ZIF‐8 nanohybrids (Figure 4C). These findings indicate that the Au@ZIF‐8 nanohybrids exhibit high biocompatibility at this concentration, supporting their potential for safe use in biomedical applications. Further, both AuNPs and ZIF‐8 individually encapsulated within GelMA hydrogels have previously been shown to have excellent biocompatibility and bone regeneration potential in vivo, respectively.^[^ ^44^, ^45^ ^]^ Taken together, in vitro data and previously published literature of similar material suggest that Au@ZIF‐8 nanohybrids encapsulated within a polymeric network exhibit biocompatible and safe behavior in vivo.

### 3.6 Osteogenic Differentiation Ability

To assess osteogenic mineral deposition induced by the Au@ZIF‐8 nanohybrid, Alizarin Red staining was performed, confirming significant calcium mineral deposition. The results showed that the Au@ZIF‐8 nanohybrid promoted substantially higher calcium deposition compared to both the control and Au@ZIF‐8 (‐) groups, indicating an enhanced osteogenic differentiation process (Figure 4D). Additionally, Figure 4E showed the quantitative analysis of ARS intensity, demonstrating a significant increase in mineral deposition with Au@ZIF‐8 nanohybrids. This enhancement suggests that the sustained release of zinc ions effectively promote the bone regeneration process. Furthermore, Figure 4F presented the expression levels of osteogenic‐related genes using RT‐qPCR. The results demonstrated that the presence of ZIF‐8 nanoparticles led to an upregulation of key osteogenic markers, including OCN and OPN, which showed significant increases. RUNX‐2 and BMP‐2 exhibited an increasing trend, suggesting a potential for promoting osteogenesis, further supporting the nanohybrid's role in enhancing osteogenic differentiation.

## 4 Conclusion

The synthesis of Au@ZIF‐8 nanohybrids was successfully accomplished, effectively entrapping AuNPs within the ZIF‐8 framework without compromising its structure. Various characterization techniques confirmed that the nanohybrid retained the hexagonal morphology and crystalline structure of pure ZIF‐8, with AuNPs stably integrated. The nanohybrid exhibited strong photothermal properties under NIR irradiation, leading to significant temperature increases and enhanced zinc ion release. This photothermal effect, combined with the bactericidal properties of zinc ions, enabled the nanohybrid to nearly eradicate MRSA bacteria. Additionally, the Au@ZIF‐8 nanohybrid demonstrated excellent biocompatibility and significantly promoted osteogenic differentiation, making it a promising material for applications in photothermal therapy, antibacterial treatment, and bone regeneration. The Au@ZIF‐8 nanohybrids show great capability in promoting bone repair, enhancing orthopedic implant integration, and combating antibiotic‐resistant bacterial strains without relying on traditional antibiotics. These qualities position Au@ZIF‐8 nanohybrids as a highly promising therapeutic biomaterial for future clinical applications. However, we acknowledge the critical importance of in vivo verification to fully validate the therapeutic efficacy and long‐term safety of our approach. As such, we have prioritized in vivo studies as a key focus for future research to comprehensively assess the clinical potential of this innovative biomaterial. Taken together, its potential to promote bone repair, enhance orthopedic implant integration, and ability to combat antibiotic resistant bacterial strains without the use of any antibiotics, make Au@ZIF‐8 nanohybrids a compelling new therapeutic biomaterial for future clinical use.

## Conflict of Interest

The authors declare no conflict of interest.

## Supporting information



Supporting Information

## Data Availability

The data that support the findings of this study are available in the supplementary material of this article.
