# Supplementary material for: NIR‐Responsive ZIF‐8 Metal‐Organic Framework Nanohybrids with Photothermal, Antimicrobial, and Osteoinductive Properties to Prevent Implant Infection
Source: Macromol Biosci. 2025 Feb 27;25(5):2400594. doi: 10.1002/mabi.202400594 (PMC12080314; doi:10.1002/mabi.202400594)
Supplement: Supplementary file 1 — Supporting Information [file MABI-25-2400594-s001.pdf]

Macromolecular Bioscience

Supporting Information

for *Macromol. Biosci.*, DOI 10.1002/mabi.202400594

NIR-Responsive ZIF-8 Metal-Organic Framework Nanohybrids with Photothermal, Antimicrobial, and Osteoinductive Properties to Prevent Implant Infection

*Cho-E Choi, Yasmeen Shamiya, Wei Luo and Arghya Paul**

Supporting Information

NIR-Responsive ZIF-8 Metal Organic Framework Nanohybrids with Photothermal, Antimicrobial and Osteoinductive Properties to Prevent Implant Infection

*Cho-E Choi, Yasmeen Shamiya, Wei Luo, and Arghya Paul**

C. Choi
Department of Chemical and Biochemical Engineering, The University of Western Ontario, London, ON N6A 5B9, Canada

Y. Shamiya,
Department of Chemistry, The University of Western Ontario, London, ON N6A 5B9, Canada

W. Luo
School of Biomedical Engineering, The University of Western Ontario, London, ON N6A 5B9, Canada Y. Shamiya, Department of Chemistry, The University of Western Ontario, London, ON N6A 5B9, Canada

Prof. A. Paul
Department of Chemical and Biochemical Engineering, School of Biomedical Engineering, Department of Chemistry, The Centre for Advanced Materials and Biomaterials Research, The University of Western Ontario, London, ON N6A 5B9, Canada
E-mail: arghya.paul@uwo.ca

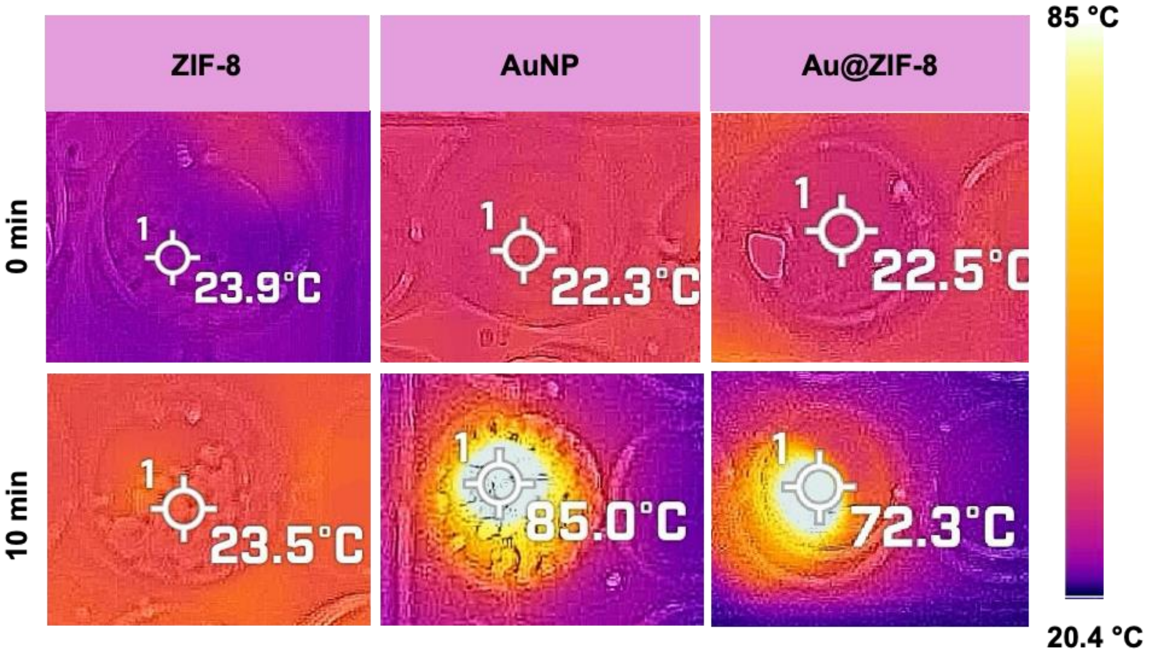

Supplementary Figure S1. Temperature variations after 10 minutes of NIR irradiation under dried conditions for nanoparticles.

Photothermal conversion efficiency

The photothermal conversion efficiencies (η) of ZIF-8 and Au@ZIF-8 were determined following the protocol described by Roper *et al.*[1–3]

$$\eta = \frac{hs(T_{\max} - T_{\text{surr}}) - Q_0}{I(1 - 10^{-A_\lambda})} \qquad (1)$$

Where h (mW/(m^2 ·°C)) is heat transfer coefficient, s (m^2) is the surface area of the container, and T_{max} is the maximum temperature, T_{surr} is the surround temperature. The maximum temperature (T_{max}) of the solution of the Au@ZIF-8 for 300 s was 63.4 °C and surround temperature (T_{Surr}) was 36.5 °C. So, the temperature change (T_{max}−T_{surr}) of the solution of the Au@ZIF-8 was 26.9 ± 0.4 °C (*n = 3*). The Q_o (mW) expresses the heat from light absorbed by the cuvette sample walls itself and it was measured to be 21.21 mW independently using a quartz cuvette cell containing aqueous samples without Au@ZIF-8 nanohybrid ($m_{cuvette}$ = 0.5 g, $C_{p,cuvette}$ = 3.03 J/g°C). I is the incident laser power (1 W) and A_λ of Au@ZIF-8 (2 mg/mL) is the absorbance (0.55) at 808 nm.

To get the hS, a dimensionless driving force temperature, θ is introduced as follows:

$$\theta = \frac{T - T_{surr}}{T_{max} - T_{surr}} \qquad (2)$$

and a sample system time constant τ_s,

$$\tau_s = \frac{\sum_i m_i C_{p,i}}{hS} \qquad (3)$$

where the i terms $miCp,i$ are products of mass and heat capacity of sample components (Au@ZIF-8 suspension, sample cell).

According to the following expression:

$$t = -\tau_s \ln \theta \qquad (4)$$

where, t represents time (s), τ_s for Au@ZIF-8 was determined to be 140.28 s. Additionally, m is 0.5 g, and Cp is 3.03 J/g°C. Therefore, according to Equation (3), hs was calculated to be 10.7 mW/°C for Au@ZIF-8. Thus, substituting according to values of each parameter to Equation (1), the 808 nm laser photothermal conversion efficiency (η) of the Au@ZIF-8 can be calculated to be 37.1%. The τ_s of ZIF-8 was determined from 300 s of cooling phase and calculated to be 61.756 s, hs is deduced to be 24.5 mW/°C (**Figure S2A and S2B**). The absorbance of the ZIF-8 (2 mg/mL) at 808 nm is 0.348, then the photothermal conversion efficiency (η) of the ZIF-8 can be calculated to be 12.2%.

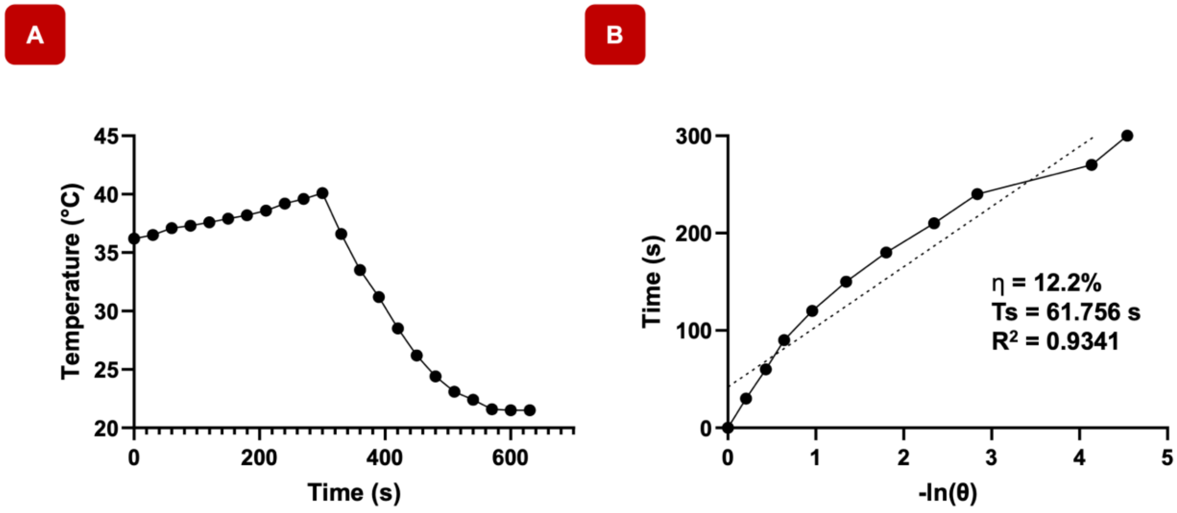

Supplementary Figure S2. A) Photothermal effect of the irradiation of the aqueous solution of the ZIF-8 (2 mg/mL) with the NIR laser (808 nm, 1.0 W/cm^2) measured at 37 °C, in which the irradiation lasted for 300 s, and then the laser was shut off. B) Linear time data versus -ln(θ) obtained from the cooling period from A.

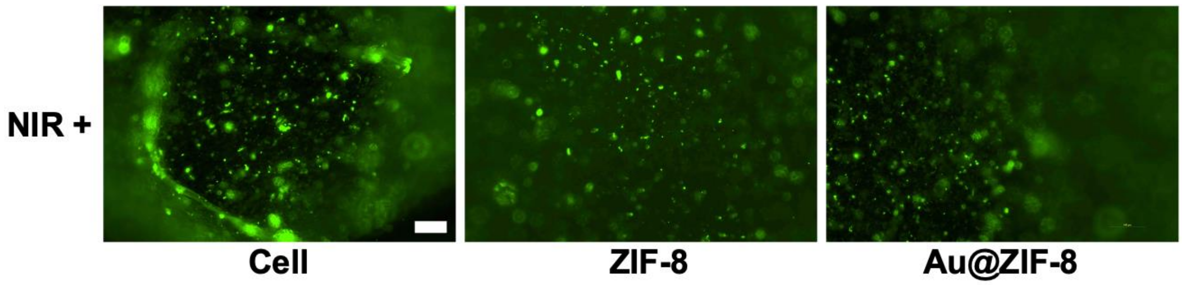

Supplementary Figure S3. Calcein AM images reveals viable cells after 5 minutes of NIR irradiation.

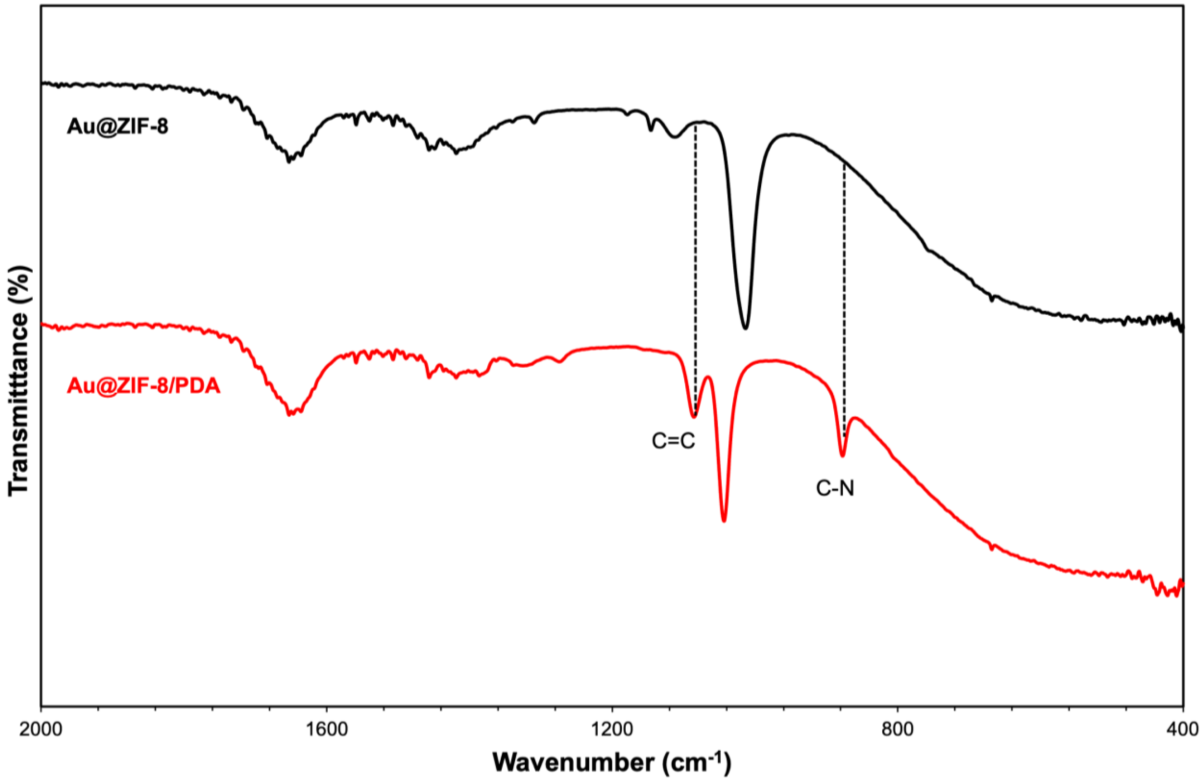

Supplementary Figure S4. The FTIR spectra of Au@ZIF-8 and Au@ZIF-8/PDA reveal distinct PDA peaks, highlighting successful conjugation of PDA to Au@ZIF-8. FTIR analysis further confirms the successful binding of Au@ZIF-8 to form Au@ZIF-8/PDA nanohybrids for coating the Ti-disc. Compared to Au@ZIF-8, the FTIR spectrum of Au@ZIF-8/PDA exhibits two additional peaks at 1088 cm^{-1} and 878 cm^{-1}, corresponding to the C=C and C-N stretching vibrations of PDA.[4,5] These findings indicate that dopamine underwent oxidation, leading to the formation of PDA, which is consistent with the conjugation process.[4,5]

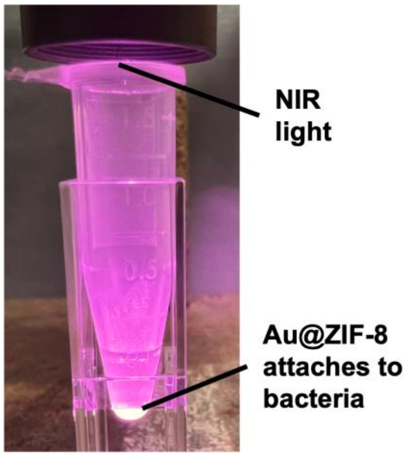

Supplementary Figure S5. Photograph of Au@ZIF-8 in the aqueous solution under light irradiation (808 nm, 1 W/cm^2).

References

[1] D. K. Roper, W. Ahn, M. Hoepfner, *J. Phys. Chem. C* **2007**, *111*, 3636.

[2] Z. Jin, D. Chen, P. Zhao, Y. Wen, M. Fan, G. Zhou, Y. Wang, Q. He, *Theranostics* **2020**, *10*, 1861.

[3] X. Liu, B. Li, F. Fu, K. Xu, R. Zou, Q. Wang, B. Zhang, Z. Chen, J. Hu, *Dalt. Trans.* **2014**, *43*, 11709.

[4] Z. Shan, H. Fan, Q. Tian, X. Jia, H. Song, *Tribol. Int.* **2024**, *192*, 109303.

[5] M. He, Y. Zhang, Y. Wang, X. Wang, Y. Li, N. Hu, T. Wu, F. Zhang, Z. Dai, X. Chen, H. Kita, *Sep. Purif. Technol.* **2021**, *275*, 119109.
